# Plasma Levels of Aromatase, Cathepsin S and Matrix Metalloproteinase 1 in Renal Cell Carcinomas: Implications for Tumor Progression and Diagnostic Value

**DOI:** 10.3390/cancers18020283

**Published:** 2026-01-16

**Authors:** Tomasz Guszcz, Anna Sankiewicz, Ewa Gorodkiewicz

**Affiliations:** 1 Department of Urology, Hospital of the Ministry of Interior and Administration in Bialystok, Fabryczna 27, 15-471 Bialystok, Poland; tomasz.guszcz@o2.pl; 2 Bioanalysis Laboratory, Faculty of Chemistry, University of Bialystok, Ciolkowskiego 1K, 15-245 Bialystok, Poland

**Keywords:** SPRi biosensor, clear cell renal cell carcinoma, aromatase, cathepsin S, matrix metalloproteinase 1

## Abstract

**Background/Objectives**: Kidney cancer (RC) is a significant global health burden. Renal cell carcinoma (RCC) is the most common form of kidney cancer. Its predominant histological subtype is clear cell renal cell carcinoma (ccRCC), which is frequently diagnosed at an advanced local stage or with metastases. Detecting cancer at an early stage significantly increases the likelihood of a cure; therefore, research on new markers and a thorough understanding of tumor biology are essential. This study investigated the significance of aromatase (ARO), cathepsin S (CTSS), and matrix metalloproteinase 1 (MMP-1) as potential biomarkers in ccRCC. **Methods**: ARO, CTSS, and MMP-1 concentrations in plasma were determined using SPRi biosensors. Appropriate antibodies were used as biorecognition molecules in the biosensors. The samples analyzed came from 60 patients with histopathologically confirmed clear cell renal cell carcinoma (ccRCC) and from 26 patients diagnosed with chronic cystitis or benign prostatic hyperplasia (BPH). **Results**: A statistically significant increase (*p* < 0.00001) in the concentration of all proteins compared with the control samples was observed at the T3–T4 stage. The ARO concentration was already statistically significantly higher at the T1–T2 stage (*p* < 0.00001). The ROC curve for aromatase demonstrated high sensitivity and specificity for detecting ccRCC, with a cut-off point of 7.53 ng mL^−1^. A moderate positive correlation was also found between the concentrations of the three tested substances in renal cancer, which may indicate potential interactions in the tumor’s pathogenesis. **Conclusions**: SPRI testing has been shown to be an alternative to standard methods for detecting potential ccRCC markers. The biosensors used in the study can simultaneously determine ARO, CTSS, and MMP-1. The results obtained suggest the potential importance of these proteins in the development of ccRCC, and our work proposes a new diagnostic technique that may aid in the diagnosis of ccRCC.

## Simple Summary

Kidney cancer (RC) is a prevalent malignant tumor. The main type of kidney cancer is renal cell carcinoma (RCC). Clear cell renal cell carcinoma (ccRCC) is a major histological subtype and is often detected at a locally advanced stage or with distant metastases. In this study, plasma concentrations of aromatase (ARO), cathepsin S (CTSS), and matrix metalloproteinase 1 (MMP-1) were determined in a control group and a group of renal cell carcinoma patients using SPRi biosensors. We examined how the presence of ccRCC affects the concentrations of individual proteins in blood plasma. Levels of the three tested substances increased with tumor stage. CTSS and MMP-1 were significantly elevated in T3–T4 lesions, while aromatase was substantially elevated in T1–T2 and T3–T4.

## 1. Introduction

Kidney cancer is a very common malignant tumor, ranking sixth among new cancer diagnoses in men and ninth in women; the highest incidence occurs in Western countries [1]. In 2023, there were 81,000 new cases of kidney cancer in the United States [2]. In Europe, there were 138,611 new cases in 2020 [3]. The main type of kidney cancer is renal cell carcinoma (RCC), accounting for approximately 90% of all malignant tumors of this organ. Among the different types of renal cell cancer, the main ones are clear cell (ccRCC—between 70% and 80% of all RCC), papillary (pRCC—between 5% and 10% of all RCC), and chromophobe (chrRCC—between 3% and 5% of all RCC) [4]. Clear cell renal cell carcinoma (ccRCC) is a renal cortical tumor originating from the proximal tubular epithelium of the kidney. It is typically a solitary, unilateral lesion with high rates of metastasis and recurrence. The best-known risk factors for ccRCC are lifestyle-related, and include smoking, obesity, hypertension, and metabolic syndrome. In ccRCC, changes in the von Hippel–Lindau (VHL) tumor suppressor gene located on chromosome 3p25.3 are also observed. VHL gene mutations, chromosome 3p deletions, or VHL gene methylations are found in most clear cell renal cell carcinomas. Loss of VHL activity mimics hypoxic conditions by blocking the proteasomal degradation of HIF-α, leading to its accumulation and triggering signals that inhibit apoptosis. The tumor consequently exhibits robust angiogenic properties [5]. Renal cell carcinoma remains asymptomatic for a long time and is often diagnosed accidentally. In more than half of patients, a renal mass is diagnosed during an abdominal ultrasound performed for various, often nonspecific, reasons or symptoms, without an initial suspicion of kidney cancer. A triad of symptoms, consisting of gross hematuria, flank pain, and a palpable abdominal mass, usually indicates an advanced stage [6]. Currently, there is no specific marker for diagnosing kidney cancer, and therefore diagnosis relies on imaging studies (ultrasound, CT, MRI) and basic laboratory tests (urine and blood tests). The most desirable biomarkers are biomolecules present in urine or blood, due to the minimally invasive nature of sampling. Thus, identifying dependable biomarkers that can facilitate the early detection of ccRCC is imperative. The disparities in the incidence of RCC between women and men may indicate that sex hormone (SH)-dependent pathways have an impact on its etiology and pathophysiology [7]. Aromatase (ARO) (also called CYP19A1) is an enzymatic complex responsible for converting androgens into estrogens. It belongs to the oxidoreductase class of the cytochrome P450 family. Aromatase catalyzes the demethylation of carbon 19 in androgens, causing their aromatization into 18-carbon estrogens. On the gonadal level, aromatase is expressed in both ovaries and testes. Aromatase activity has also been reported in many other tissues and cells (the adrenal cortex, in adrenocortical tumors, in the parietal cells of the gastric mucosa, human osteoblasts, stromal cells, and adipocytes) [8]. The cysteine cathepsin family plays a crucial role in multiple processes essential for tumor growth and progression, including angiogenesis, invasion, and metastasis. Among them, cathepsin S (CTSS) deserves special attention. CTSS possesses features that differentiate it from other cysteine cathepsins. Unlike most family members, CTSS remains functional at neutral and acidic pH levels. CTSS is strongly expressed in malignant tissues [9]. Correlations with clinicopathological features of tumors, as well as with tumor invasion of other cells, have been observed [10]. Peng et al. demonstrated that the concentration of cathepsin S had a significant positive correlation with the risk of papillary renal cell carcinoma (pRCC) [11]. The tumor microenvironment (TME) can serve both as a barrier and as an opportunity for cancer progression and treatment. Modifications to the microenvironment occur under the influence of various factors. The cytokines and chemokines which are released by cells in the TME mediate the complex communication that affects tumor growth and immune responses. CTSS has been demonstrated to be a crucial mediator within the inflammatory microenvironment of tumors. Changes in the TME are associated with the remodeling and degradation of the extracellular matrix (ECM) basement membrane [12]. Previous studies have demonstrated changes in tissue and serum levels of collagen IV, laminin-5, and fibronectin in ccRCC [13]. The extracellular matrix degradation is caused by enzymes such as matrix metalloproteinases (MMPs). MMP-1, as a key member of the MMP family, plays an essential role in tumor growth, differentiation, angiogenesis, migration, invasion, and immune surveillance of cancer. MMP-1 degrades fibrillar collagen, a key component of the ECM and basement membranes, essential for tumor cell invasion and metastasis. High MMP-1 activity is linked to tumor progression and metastasis in various cancers [14,15,16], including renal cell carcinoma [17]. Our work aimed to assess plasma concentrations of ARO, CTSS, and MMP-1, using SPRi biosensors in a control group and in a group of patients with renal cell carcinoma. We examined the effect of the ccRCC stage on the concentration of individual proteins in the plasma. To assess the diagnostic usefulness of ARO, CTSS, and MMP-1 in ccRCC, we performed ROC analysis. Moreover, we evaluated the strength of the intercorrelation between the studied proteins.

## 2. Materials and Methods

### 2.1. Reagents

“The following reagents were used for the tests: aromatase and anti-aromatase rabbit polyclonal antibody (Lucerna-Chem AG, Luzern, Switzerland), cathepsin S and anti-cathepsinS rat monoclonal antibody (R&D system, Minneapolis, MN, USA), matrix metalloproteinase-1 (Cloud-Clone Corp, Katy, TX, USA), monoclonal rabbit anti-matrix metalloproteinase-1 antibody (Ray Biotech, Norcross, GA, USA). In addition, also used were: EDC (N-ethyl-N′-(3-dimethylaminopropyl) carbodiimide) (Sigma, Steinheim, Germany), NHS (N-hydroxysuccinimide) (Aldrich, Steinheim, Germany), carbonate buffer pH = 8.5, 2-aminoethanethiol (cysteamine) (Aldrich, Steinheim, Germany), human albumin (Sigma, Steinheim, Germany), absolute ethyl alcohol 99.8% (POCh, Gliwice, Poland), HBS-ES solution (pH = 7.40, 0.01 M HEPES, 0.15 M sodium chloride, 0.005% Tween-20, 3 mM EDTA) and PBS (pH = 7.40, phosphate-buffered saline) (BIOMED, Tokyo, Japan)”. A glass plate with a gold layer, constituting the basis of the biosensor, was obtained from Ssens (Ssens, Enschede, The Netherlands).

### 2.2. Biological Material and Its Preparation

Patients with clear cell renal cell carcinoma hospitalized at the J. Sniadecki Provincial Hospital in Białystok (Poland) were enrolled in the study. The study cohort was stratified into two groups: patients with ccRCC and controls. Plasma was collected from patients with renal carcinoma diagnosed by computed tomography. Definitive diagnosis was confirmed by histopathological evaluation of tumor samples, which were taken following either total or partial nephrectomy. The patient cohort was divided into subgroups: stages T1–T2 and stages T3–T4 (according to the TNM classification for renal cancer). The control group comprised patients with benign prostatic hyperplasia (diagnosed by ultrasonography) or chronic cystitis (persistent bladder inflammation confirmed by histopathological assessment). The control group consisted of patients with benign prostatic hyperplasia (diagnosed by ultrasound) or chronic cystitis (persistent cystitis confirmed by histopathological examination). Each patient had undergone a CT scan or ultrasound within the three months preceding enrollment in our study’s control group. Based on these scans, no malignant lesions were detected, and they had no history of malignancy. The study participants’ clinical characteristics are presented in Table 1.

Ethical approval for this study was obtained from the Bioethics Committee of the Medical University of Białystok, Poland (approval number R-I-002/461/2016). Written informed consent was obtained from all participants.

Venous blood was collected from the antecubital vein into vacuum tubes containing ethylenediaminetetraacetic acid (EDTA). Samples were centrifuged at 2000× *g* for 10 min at 4 °C. The plasma was collected from above the sediment and immediately frozen and stored at −70 °C. For the determination of aromatase, cathepsin S, and matrix metalloproteinase 1 concentrations, the samples were thawed immediately before analysis and diluted with phosphate-buffered saline (PBS) to ensure that analyte concentrations lay within the linear range of the calibration curve.

### 2.3. Procedure for Quantifying ARO, CTSS and MMP-1

**Figure 1:**
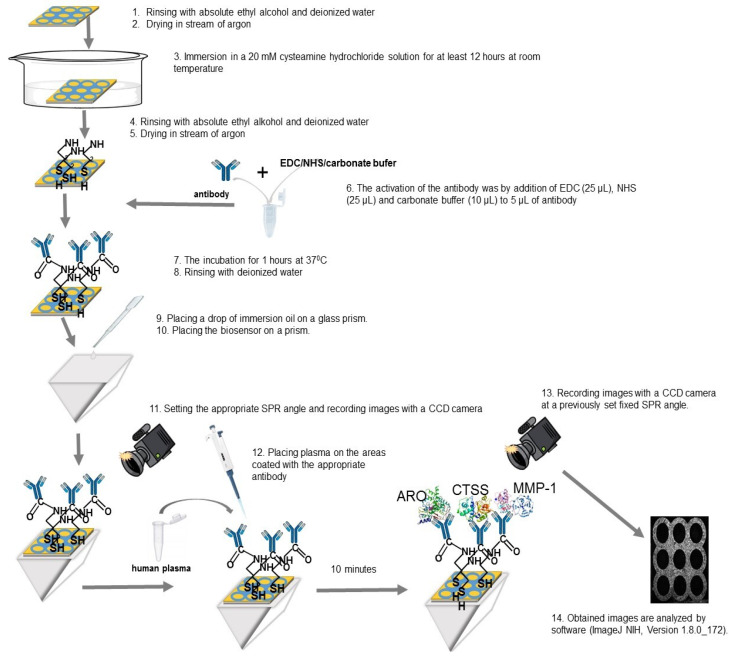
Diagram of the protocol for quantifying aromatase (ARO), cathepsin S (CTSS), and matrix metalloproteinase (MMP-1).

Quantitative determinations of ARO, CTSS, and MMP-1 were made using previously developed SPRi biosensors. The protocols for measuring these biomolecules are described in previous papers [18,19,20]. The protocol diagram is shown in Figure 1. Briefly, the biosensor consists of a glass plate with a spattered layer of titanium (1 nm) and gold (50 nm). The gold surface was modified by creating a thiol (cysteamine) layer, on which the bioreceptors (antibody) were then immobilized, capturing the corresponding proteins. The bioreceptors for ARO, CTSS, and MMP-1 were a rabbit polyclonal antibody (concentration = 20 ng mL^−1^), a rat monoclonal antibody (concentration = 20 ng mL^−1^), and a rabbit monoclonal antibody (concentration = 170 ng mL^−1^), respectively. The selectivity of the biosensors used was determined during the validation stage of each and expressed in terms of recovery.

SPRi measurements were performed using an SPRi apparatus constructed in the Bioanalysis Laboratory at the Faculty of Chemistry, University of Białystok. Details of the construction are described in previous publications [18,19,20]. SPRi is a label-free optical technique, susceptible to changes in mass occurring on the metallic surface. As a result of the adsorption of successive biomolecules, changes in mass and changes in the light refractive index occur. The signal is measured and converted by a CCD camera that records the reflected light. The image is recorded by a camera before and after the ligand–analyte interaction. The measurement results are presented in the form of a 2D image. The images obtained are analyzed by software (ImageJ NIH, Version 1.8.0_172). Based on the measured signals, concentrations are determined from calibration curves prepared using standard solutions of the tested proteins prior to measurement. Appendix A.

### 2.4. Statistical Analysis

ARO, CTSS, and MMP-1 levels were examined in two groups of patients: patients with clear cell renal cell carcinoma, and patients with benign prostatic hyperplasia or chronic cystitis. The ccRCC cohort was divided into subgroups: stages T1–T2 and stages T3–T4. The significance of differences between the groups was evaluated after testing for normal distribution using the Shapiro–Wilk test, followed by the Kruskal-Wallis test for comparisons across multiple groups (control, T1–T2, and T3–T4). The level of statistical significance was set at *p* < 0.05. The Dunn–Bonferroni test was used after the Kruskal–Wallis test to perform multiple pairwise comparisons and identify which specific groups were significantly different from each other. The method used to determine the most effective threshold for classification and to select an optimal cut-off value for determining the presence or absence of a disease was based on the receiver operating characteristic (ROC) curve. Intercorrelations between ARO, CTSS, and MMP-1 in human serum were calculated using Spearman’s rank correlation coefficient. Statistical analysis was performed using the commercial software PQStat (PQStat Software (2022), PQStat v.1.8.4, Poznań, Poland).

## 3. Results

This section will be divided by subheadings. Each subsection will provide a concise and precise description of the experimental results, their interpretation, and the experimental conclusions that can be drawn.

### 3.1. Changes in Plasma Concentration

**Figure 2:**
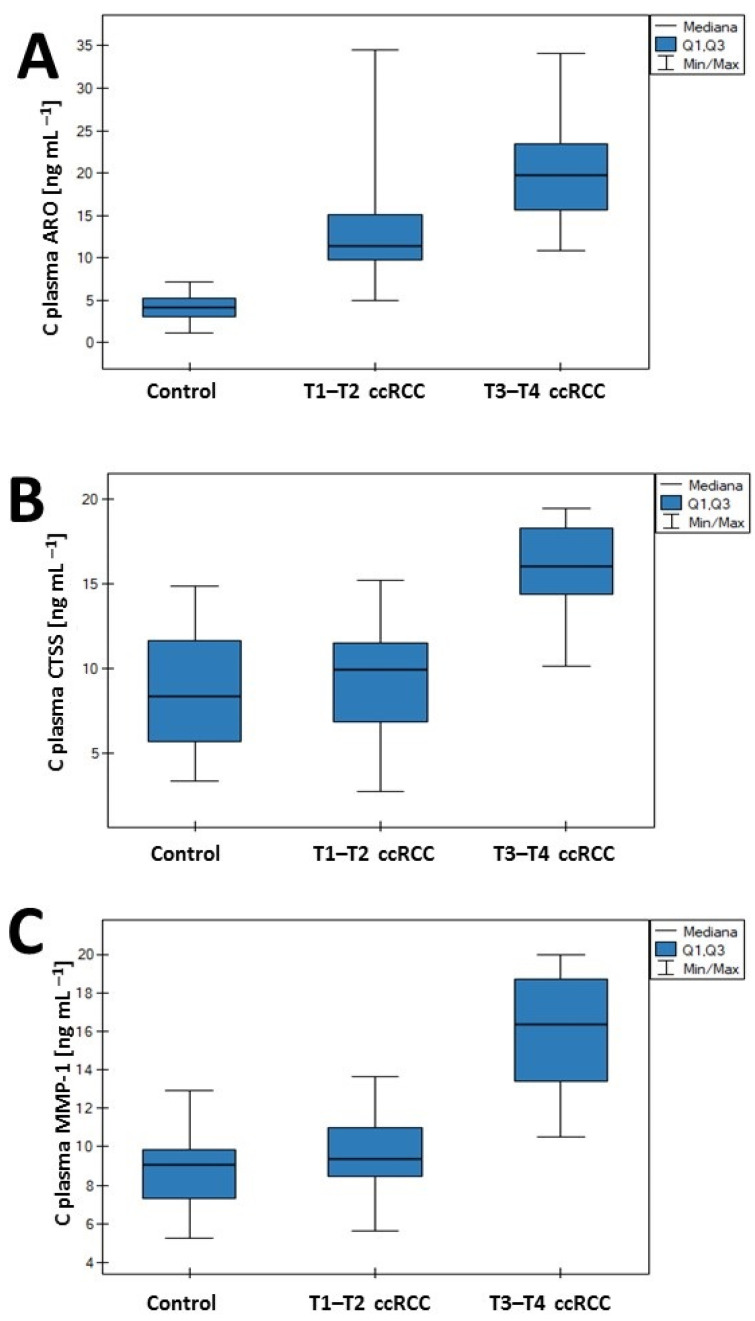
Concentrations and post hoc test results of (**A**) aromatase (ARO), (**B**) cathepsin S (CTSS), and (**C**) matrix metalloproteinase (MMP-1).

Serum concentrations of ARO, CTSS, and MMP-1 were analyzed. Participants were classified into three groups: control, stage T1–T2, and stage T3–T4. Aromatase plasma levels were significantly elevated in patients with ccRCC compared with the control group. Significant differences were also observed between the T1–T2 and T3–T4 stages. In contrast, statistically significant differences in CTSS and MMP-1 concentrations were found only between the control group and patients with stage T3–T4 of the disease (Figure 2). The concentration values obtained are provided in Table A2 (Appendix A).

### 3.2. ROC Analysis

**Figure 3:**
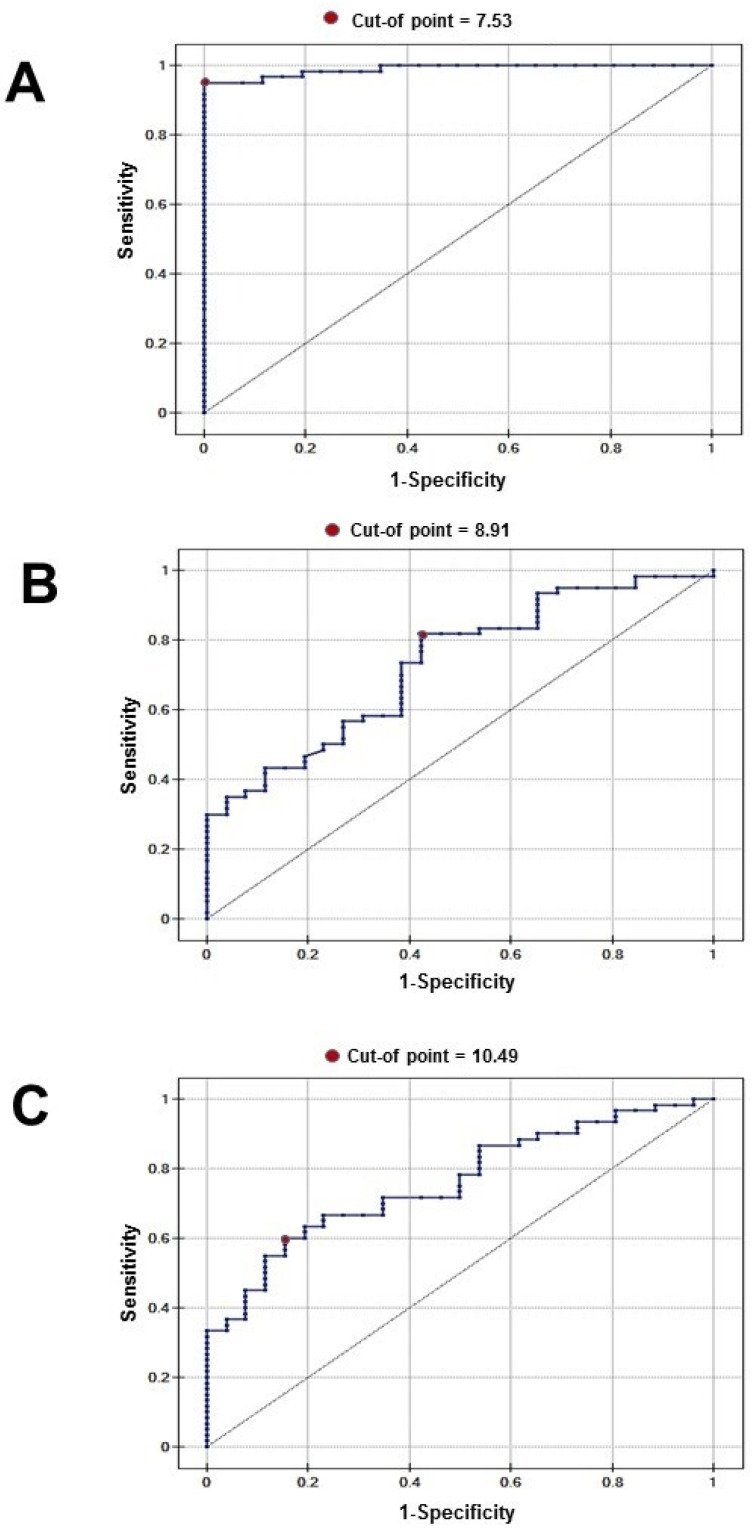
ROC curve for (**A**) plasma aromatase (ARO), (**B**) plasma cathepsin S (CTSS), and (**C**) plasma matrix metalloproteinase-1 (MMP-1) ccRCC vs. control.

Receiver operating characteristic (ROC) curve analysis was performed, and optimal diagnostic cut-off values for the plasma concentrations of the examined biomarkers in renal cancer were established. These values enabled discrimination between healthy controls and renal cancer patients. The corresponding ROC curves are presented in Figure 3. Red dots denote the cut-off points, and the essential diagnostic parameters are summarized in Table 2.

### 3.3. Correlation

Intercorrelations between ARO, CTSS, and MMP-1 in human serum from patients with clear cell renal cell carcinoma (ccRCC) were examined. The estimated intercorrelations among proteins are presented in Table 3. A moderate positive correlation was observed between all studied variables. Spearman’s rank correlation coefficient (rs) ranged from 0.46 to 0.58.

## 4. Discussion

Clear cell carcinoma is the most common form of renal cancer; unfortunately, it is often detected at a locally advanced stage or with distant metastases. It exhibits poor sensitivity to conventional radiotherapy and chemotherapy [21]. Detecting cancer at an early stage significantly increases the likelihood of a cure; therefore, research on new markers and a thorough understanding of tumor biology are essential. The aim of this study is to examine the plasma concentrations of three substances: cathepsin S (CTSS), aromatase (ARO), and matrix metalloproteinase 1 (MMP-1). To our knowledge, simultaneous determination of these three parameters in renal cancer has not been reported previously. The literature on the role of these substances in renal cancer is scarce. The expression and activity of cathepsin S have been shown to be elevated in several cancers, including breast [22], colon [23], and prostate [24]. Peng et al. demonstrated that the concentration of cathepsin S had a significant positive correlation with the risk of papillary renal cell carcinoma (pRCC) [12]. A study by Wang determined that cathepsin S expression was higher in ccRCC tissue than in surrounding tissue, and interestingly, decreased with stage progression [10]. In our study, however, plasma cathepsin S concentrations were found to be slightly elevated in stage T1–T2 compared with the control group (9.96 vs. 8.38 ng mL^−1^), and significantly more elevated in the T3–T4 group (16 vs. 8.38 ng mL^−1^). Available studies suggest that differences in the incidence of kidney cancer in women and men may be related to the potential influence of sex hormones on the carcinogenesis process [7]. Kirma et al. [25] indicate that aromatase, by increasing estrogen concentration in the tumor microenvironment, may activate oncogenic genes while simultaneously deactivating tumor suppressor genes, leading to an imbalance between them and tumor proliferation. A potential role of aromatase in cancer development has been demonstrated in breast cancer [26] and bladder cancer [27]. Aromatase is often highly expressed in macrophages accompanying neoplastic lesions (TAM—tissue associated macrophages) [28]. Similarly, cathepsin S has been shown to be highly expressed in immune cells, particularly macrophages [10]. It appears that the common increased expression of both substances may have a pathophysiological basis, indicating their interdependence. According to the literature, cathepsin S activates the NF-κB pathway, leading to the increased secretion of pro-inflammatory cytokines such as IL-1β, TNF-α, and IL-6, which in turn activate aromatase expression [29,30]. Our study also demonstrated a correlation between cathepsin S and aromatase concentrations (R = 0.501, *p* = 0.00004). However, unlike cathepsin S, aromatase attained significantly higher concentrations in stages T1–T2 compared with the control group (11.45 vs. 4.08 ng mL^−1^), reaching the highest values in stages T3–T4 (19.76 ng mL^−1^). This result suggests that aromatase may be a more sensitive parameter for determining renal tumor stage than cathepsin S. The ROC curve analysis demonstrated that aromatase exhibited high sensitivity and specificity for the detection of ccRCC, with a cut-off point of 7.53 ng mL^−1^. The results were internally cross-validated, yielding an AUC value close to 0.98. We can speculate that the increase in aromatase concentration may be stimulated by even a slight increase in cathepsin S concentration and its effect on pro-inflammatory cytokines; alternatively, it may result from completely independent factors. It has been demonstrated that in uterine tissue and in endometriosis, there is significant co-expression of MMP-1 and Erβ, which may suggest estrogen-mediated regulation of MMP-1 [31]. Similar studies by Potier showed that in mesangial cells of patients with glomerulonephritis, there was a significant increase in the expression and activity of MMP-9 that was attributed to the increased expression of both estrogen receptors, ERα and ERβ [32]. This finding may be consistent with our results, where increased aromatase activity resulted in an increase in estrogen levels in the tumor microenvironment, leading to a rise in MMP-1 concentration. MMP-1 concentration was slightly higher in the group with T1–T2 lesions than in the control group, and significantly higher in the T3–T4 group (control 9.06, T1–T2 9.39, T3–T4 16.33 ng mL^−1^).

## 5. Conclusions

SPRi testing has been shown to be an alternative to standard methods for detecting potential ccRCC markers. The levels of the three tested substances increased with tumor stage and were significantly elevated in the T3–T4 group, while aromatase was substantially elevated even at the T1–T2 stage. Surprisingly, the ROC curve for aromatase demonstrated high sensitivity and specificity for detecting ccRCC, with a cut-off point of 7.53 ng mL^−1^. A correlation was also found between the concentrations of the three tested substances in renal cancer, which may indicate their potential interactions in the pathogenesis of the tumor.

The study presented here has several limitations. Due to the small sample size, particularly in the T3–T4 group, the study may have limited statistical power. Furthermore, the ROC curves for aromatase demonstrate near-perfect efficacy, which may be due to overfitting in this dataset. We recognize the need for external validation in a larger patient population. Our study is a preliminary investigation, and further studies, including urine tests, should be performed on a larger group of patients to determine the value of the tested substances as potential markers of ccRCC.

## Figures and Tables

**Table 1:** Clinical characteristics of patients.

Group	Number of Patients	Average Age	Sex
Control group:	26	67 (36–86)	F (13), M (13)
BPH (benign prostate hyperplasia)	11	71 (55–84)	F (0), M (11)
Cystitis chronica	15	64 (36–86)	F (10), M (5)
ccRCC:	60	63 (38–83)	F (27), M (33)
TNM T1-T2	33	62 (38–76)	F (12), M (27)
TNM T3-T4	27	67 (55–82)	F (12), M (15)

**Table 2:** Diagnostic efficiency of aromatase (ARO), cathepsin S (CTSS), and plasma matrix metalloproteinase-1 (MMP-1).

Fig	AUC	*p*	Sensitivity	Specificity	PPV	NPV	95% CI	Cut-Off
ARO plasma Cancer Presence (3A)	0.98	<0.0001	0.95	1	1	0.89	0.97–1.00	7.53
CTSS plasma Cancer Presence (3B)	0.73	<0.0001	0.82	0.58	0.82	0.58	0.62–0.84	8.91
MMP-1plasma Cancer Presence (3C)	0.76	0.0008	0.60	0.85	0.90	0.48	0.65–0.86	10.49

**Table 3:** Inter-correlations between ARO, CTSS and MMP-1 in human serum from patients with ccRCC (*n* = 60).

Inter-Correlation	Spearman’s Rank Correlation Coefficient (r_s_)	*p*-Value (2-Tailed)
ARO/CTSS	0.501	*p* = 0.00004
ARO/MMP-1	0.460	*p* = 0.00022
CTSS/MMP-1	0.580	*p* < 0.00001

**Figure A1:**
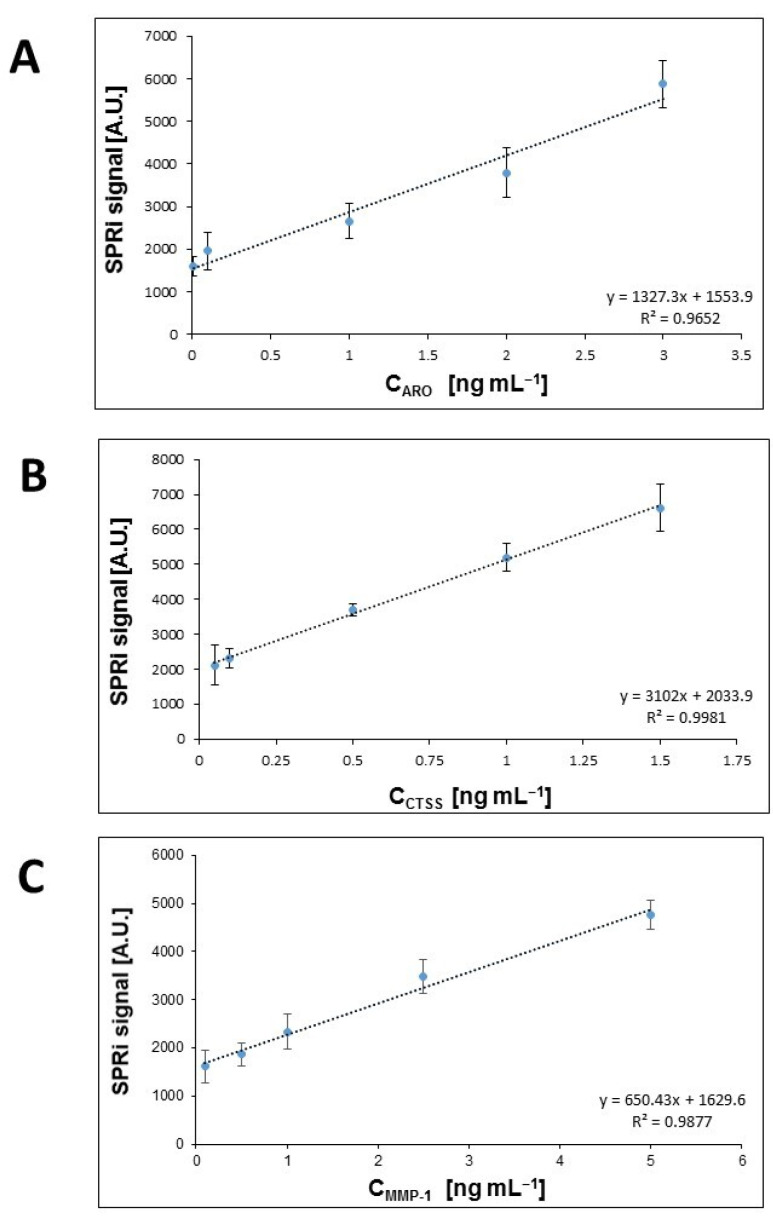
Calibration curve for determination of (**A**) plasma aromatase (ARO), (**B**) plasma Cathepsin s (CTSS), and (**C**) plasma matrix metalloproteinase (MMP-1).

## Data Availability

The data presented in this study are available upon request from the corresponding author due to legal and ethical reasons.

## Appendix A

The final SPRi signal was calculated as the difference between the signals acquired from images recorded before and after analyte interaction with the biosensor recognition element. The concentrations of ARO, CTSS, and MMP-1 in the sample were calculated from calibration curves generated immediately before the measurements. The final concentration in plasma was determined after considering the sample dilutions. The resulting calibration curves are shown in Figure A1. Details of the methods for determining these proteins have been described in earlier publications [18,19,20]. The parameters of the methods are given in Table A1.

**Table A1:** Parameters of the methods for the determination of ARO, CTSS, and MMP-1.

Method	LOD	LOQ	Linearity	CV [%]	Recovery [%]
ARO	0.09 [ng mL^−1^]	0.30 [ng mL^−1^]	0.30–5.00 [ng mL^−1^]	2.50	97–108
CTSS	0.04 [ng mL^−1^]	0.14 [ng mL^−1^]	0.14–2.50 [ng mL^−1^]	7.14	100–103
MMP-1	9.00 [pg mL^−1^]	18.0 [pg mL^−1^]	0.05–20.00 [ng mL^−1^]	2.30	82–113

**Table A2:** Protein concentrations in plasma obtained in the studied groups.

Parameter	Control Median (Range)	T1-T2 N0M0 (TNM) Median (Range)	T3-T4N0M0 (TNM) Median (Range)
Aromatase [ng mL^−1^]	4.08 (1.13–7.13)	11.45 (4.92–34.51)	19.76 (10.81–34.12)
Cathepsin S [ng mL^−1^]	8.39 (4.11–14.84)	9.97 (2.76–15.22)	16.00 (10.17–19.45)
Matrix metalloproteinase 1 [ng mL^−1^]	9.06 (5.27–12.91)	9.39 (5.65–13.63)	16.33 (10.51–19.97)
